# Comparison of Metabolic Pathways in *Escherichia coli* by Using Genetic Algorithms

**DOI:** 10.1016/j.csbj.2015.04.001

**Published:** 2015-04-09

**Authors:** Patricia Ortegon, Augusto C. Poot-Hernández, Ernesto Perez-Rueda, Katya Rodriguez-Vazquez

**Affiliations:** a Departamento de Ingeniería de Sistemas Computacionales y Automatización, IIMAS, Universidad Nacional Autónoma de México, Mexico; b Departamento de Ingeniería Celular y Biocatálisis, Instituto de Biotecnología, Universidad Nacional Autónoma de México, Cuernavaca, Morelos, Mexico; c Unidad Multidisciplinaria de Docencia e Investigación, Sisal Facultad de Ciencias, Sisal, Yucatán, UNAM, Mexico

**Keywords:** Metabolism, Genetic algorithms, KEGG database, *k-*medoids, Comparative genomics

## Abstract

In order to understand how cellular metabolism has taken its modern form, the conservation and variations between metabolic pathways were evaluated by using a genetic algorithm (GA). The GA approach considered information on the complete metabolism of the bacterium *Escherichia coli* K-12, as deposited in the KEGG database, and the enzymes belonging to a particular pathway were transformed into enzymatic step sequences by using the breadth-first search algorithm. These sequences represent contiguous enzymes linked to each other, based on their catalytic activities as they are encoded in the Enzyme Commission numbers. In a posterior step, these sequences were compared using a GA in an all-against-all (pairwise comparisons) approach. Individual reactions were chosen based on their measure of fitness to act as parents of offspring, which constitute the new generation. The sequences compared were used to construct a similarity matrix (of fitness values) that was then considered to be clustered by using a *k*-medoids algorithm. A total of 34 clusters of conserved reactions were obtained, and their sequences were finally aligned with a multiple-sequence alignment GA optimized to align all the reaction sequences included in each group or cluster. From these comparisons, maps associated with the metabolism of similar compounds also contained similar enzymatic step sequences, reinforcing the *Patchwork Model* for the evolution of metabolism in *E. coli* K-12, an observation that can be expanded to other organisms, for which there is metabolism information. Finally, our mapping of these reactions is discussed, with illustrations from a particular case.

## 1 Introduction

The study of the evolution of metabolism is fundamental to understanding the adaptive processes of cellular life, the emergence of high levels of organization (multicellularity), the diversity of cellular organization in the three major domains of life, *Archaea*, *Bacteria*, and *Eukarya*, and the complexity of the living world [4]. At present, the large scale of information derived from genomics and proteomics studies has allowed the construction of diverse databases devoted to organizing the metabolic processes, such as the KEGG [21], and MetaCyc [5]. Therefore, the information contained in these databases can be used to generate an integrative perspective on cellular functioning.

Metabolism can be considered one of the most ancient biological networks; in such a network, the nodes represent substrates or enzymes and the edges represent the relationships among them. From a global perspective, the comparative analysis of metabolic pathways aims to identify similarities and differences among them, providing insights for the identification of evolutionary events, such as enzyme recruitment and duplication events. In this regard, metabolic pathways exhibit high retention of duplicates within functional modules and a preferential biochemical coupling of reactions. This retention of duplicates may result from the biochemical rules governing substrate-enzyme–product relationships [1,8,14,19].

In this context, diverse studies have evaluated the variations among pathways, both intra- and interspecies [7,27], comparing the pathways based on their Enzyme Commission (EC) numbers and excluding information on the compounds. In addition, a method of path-and-graph matching has been proposed to query metabolic pathways based on a predefined graph, where a similarity measure based on EC numbers [30] and the distance between pathways as a combination of distances between compounds and between enzymes associated with amino acid biosynthesis networks are considered [11].

In this work, we evaluated whether there are groups of similar reactions in different pathways, which might suggest a transfer of enzymatic activities, and whether these groups can be used to define common and variable regions of an organism's metabolism. This analysis was addressed using EC numbers, coded as a succession of reaction steps. To this end the metabolic maps of the bacterium *Escherichia coli* K-12, as deposited in the KEGG database, were transformed into linear Enzymatic Step Sequences (ESS), to be compared via a genetic algorithm (GA). The sequences compared were used to construct a similarity matrix to identify groups of conserved reactions based on a *k*-medoids clustering analysis, and then a multiple-sequence alignment (MSA) GA was optimized to align all the reaction sequences included in a group. Finally, we consider our comparisons in terms of the clues they provide in reinforcing the *Patchwork Model* in the evolution of metabolism for *E. coli* K-12 and probably for other organisms beyond this bacterium.

## 2 Methods

### 2.1 Construction of Enzymatic Step Sequences (ESS)

**Fig. 1:**
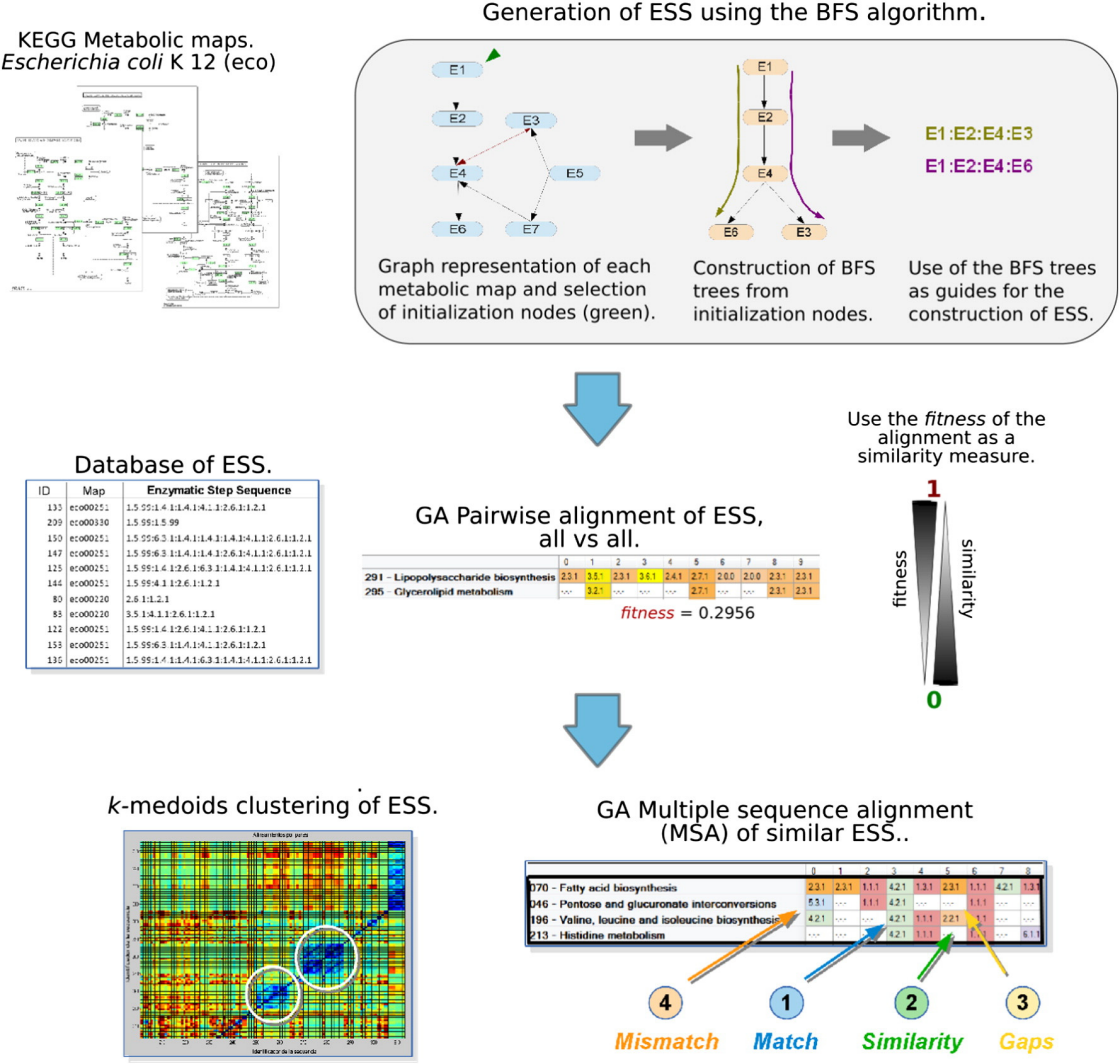
General strategy for the comparative analysis of *E. coli* K-12 metabolism. The metabolic maps from KEGG were converted to ESS by using the breadth first search (BFS) algorithm. For each map a graphical representation was created, where nodes represent enzymes and edges are product-substrate relationships. Then, a set of initialization nodes was selected (green arrowhead) as roots for BFS trees. Those trees were used as guide for ESS construction. Afterwards all the ESS were compared against each other by GA pairwise alignments. The similarities among ESS were used to conduct a clustering analysis based on the *k*-medoids algorithm. Finally, clusters of similar sequences were aligned using an MSA approach.

**Fig. 2:**
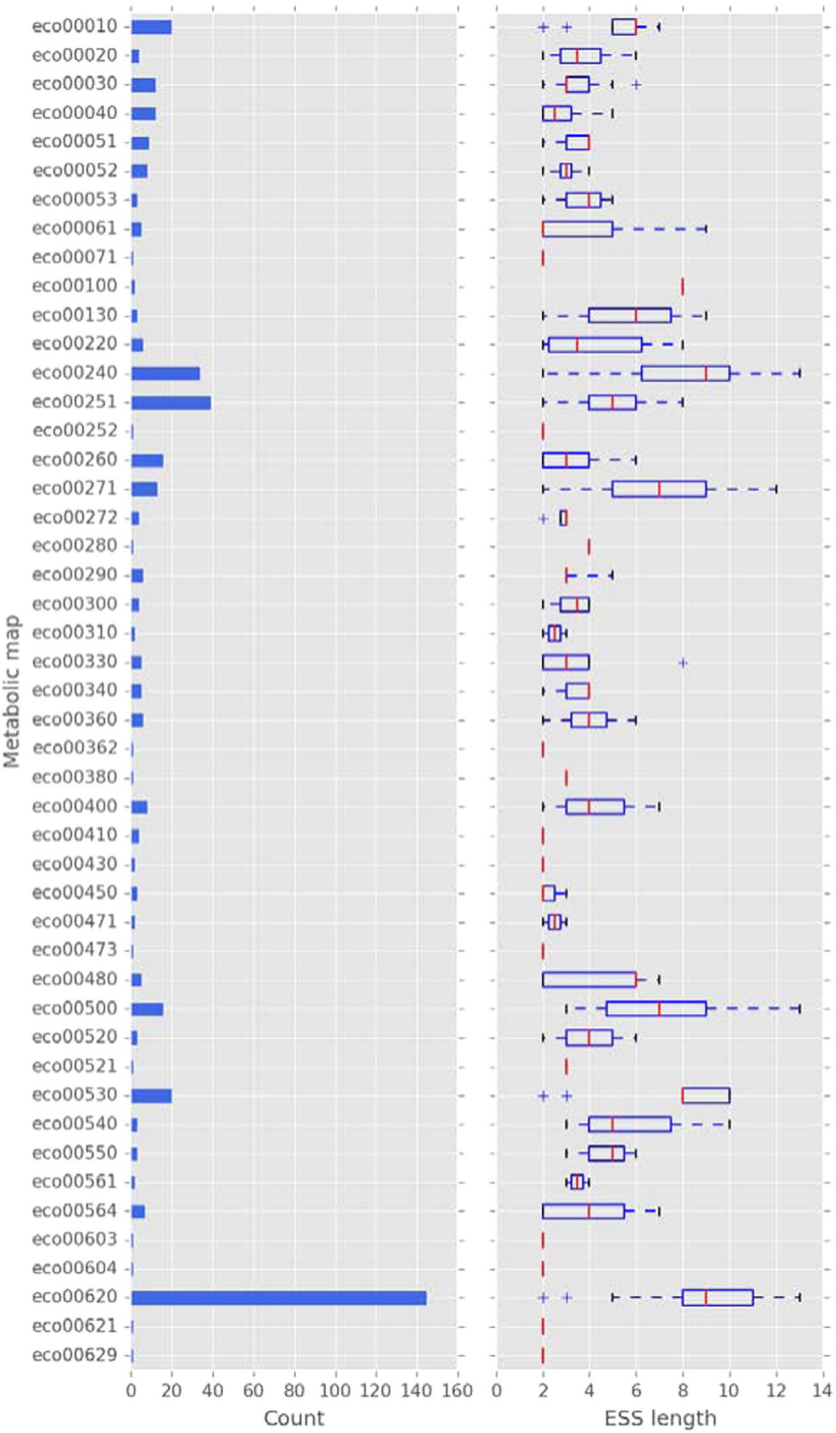
Statistics for the ESS per metabolic map. Only the 45 metabolic maps that generate at least one sequence are shown. In the left panel, the number of ESS generated by the metabolic map are shown; in the right panel, the distribution of lengths of those sequences is shown.

The KGML files (version 0.71) that describe the metabolic maps (pathways) of *E. coli* K-12 as of June 2011 were downloaded from the KEGG database (Fig. 1). Pathways were transformed into linear ESS by using the breadth-first search (BFS) algorithm [26], which infers the closer neighbor of each enzyme by considering a common compound, a substrate or product. In brief, a directed graphical representation of each metabolic map was created in which the nodes represented enzymes and the edges represented a shared substrate/product between two enzymes. This representation takes into account the reversibility of the reactions. Then, a group of BFS trees was generated for each metabolic map from a set of initialization nodes, which were used as roots. In this work, an initialization node was defined by two criteria: (i) a node whose substrate is not catalyzed by another enzyme in the metabolic map, and (ii) a node whose substrate comes from another metabolic map and has two or fewer neighbors in the graph. These criteria represent the metabolic input for each pathway; the first criterion considers the substrates not created in the same pathway, and the second one considers the connections with other pathways. Each initialization node was used as a root for the construction of a BFS tree. Thus, each tree was used as a guide for the construction of the corresponding ESS. In this way, a BFS tree creates as many ESS as the number of branches it contains. Finally, the first three levels of EC numbers are used to represent an enzyme as a string or sequence (Fig. 1). ESS constructed per metabolic map is shown in Fig. 2 and Table S1, with mean lengths ± standard deviation (SD).

### 2.2 Sequence Alignments Obtained via Genetic Algorithms

The proposed GA starts by creating a random initial population of variable-length chromosomes, which represents the potential for alternative alignments. For each iteration or generation, the population evolves by means of selection, crossover, and mutation. Here, a tournament selection that randomly selected a subset of individuals and chose the best individual of each subset was used.

### 2.3 Crossover Operator

**Fig. 3:**
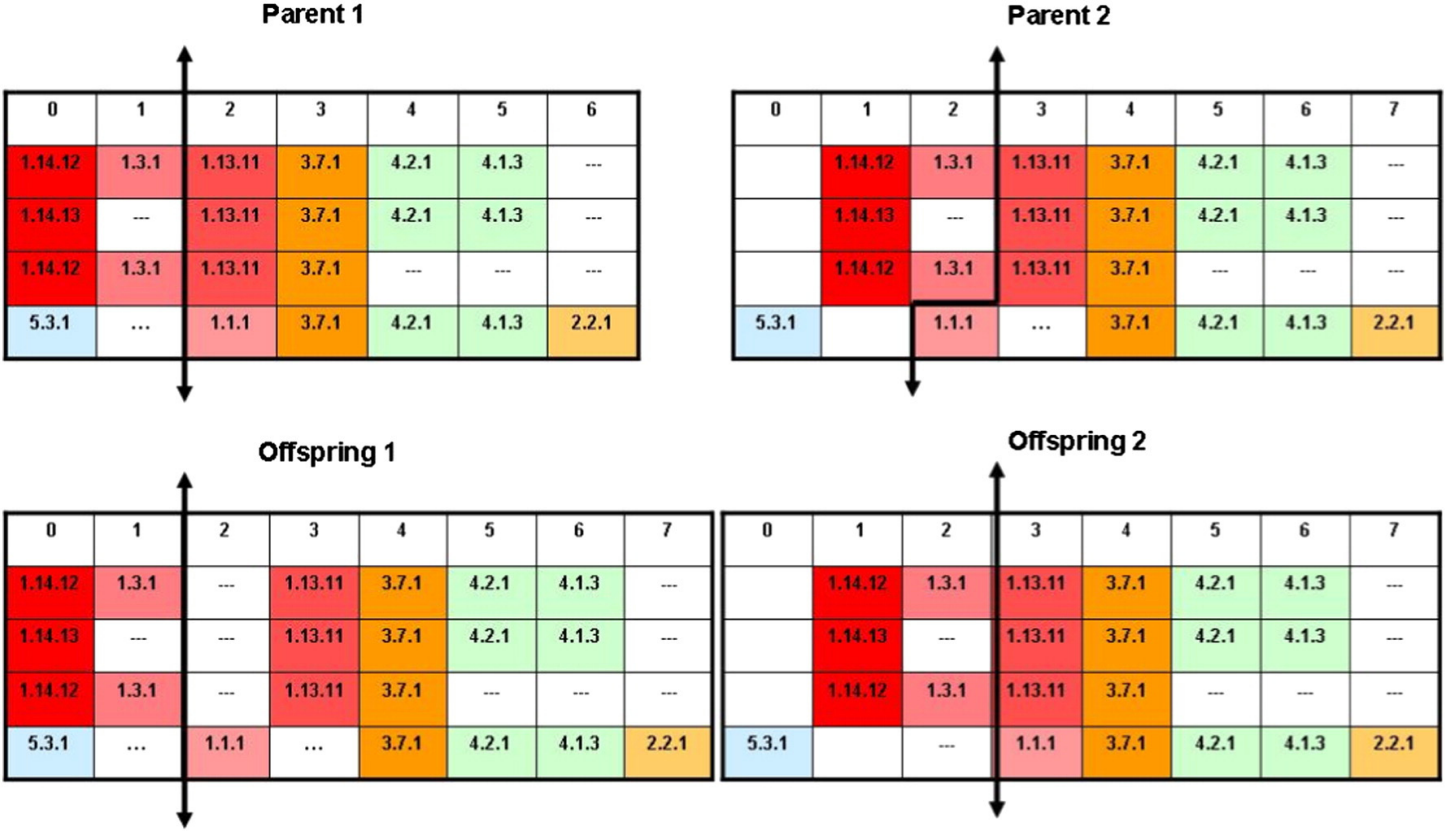
Crossover for MSA of metabolic pathways.

In this work, one-point crossovers were considered; however, it is important to note that for crossover two alternative metabolic pathway sequence alignments, they must be in general, of different sizes (i.e. have a different number of columns). Then, this operator is used to select a position in the first parent and the second parent is cut, keeping in mind the EC numbers conserved for the left side of the first parent. As shown in Fig. 3, offspring 1 is generated by combining the left of parent 1 and the right of parent 2, inserting a gap in the first column for the first three rows of the right side of parent 2. Offspring 2 is produced by combining the left side of parent 2 (inserting a gap at the end of the last row) with the right side of parent 1. Thus, the EC numbers are kept constant, and only gaps vary.

### 2.4 Mutation

A binary codification was used, where 1 represented an enzyme and 0 represented a gap. The gap insertion was highly penalized. The algorithm was designed to find the best alignment with the maximum score. Therefore, if the selected position is a gap, this can be extended or reduced in one unit with an uniform probability; on the contrary, if the selected position corresponds to an EC number, a gap is inserted.

### 2.5 Objective Function

Assessment of the quality of an alignment considers the column homogeneity, with penalizations for gap insertions and column increments. Thus, the proposed objective function (O.F.) is composed of three weighted terms, defined in Eq. (1):

(1) O.F.=0.9Homogeneity+0.05GapPenalty+0.05ColumnIncrement.

### 2.6 Homogeneity

The sum of pairs is a popular criterion for column homogeneity evaluation, as it assigns a cost to each pair of aligned codifications in each column of an alignment (substitution cost) and a cost to gap insertions (gap costs). The sum of these costs yields the global cost of the alignment. In this work, rating the grade of diversity in the elements of a given position (column) was evaluated as a measure of column homogeneity.

The EC numbers (columns in the alignment) are represented by three levels (subcolumns), evaluating the normalized entropy of each level based on Shannon's entropy. These results are weighted, giving a higher value to the first level. Gaps are considered one more symbol, and a penalty is applied to compensate for the possibility of “false homogeneity” indicated by a high number of gaps in a column. The following equations give details for the evaluation of the homogeneity of each column. Given alignment *M*, where *m_j_* is the *jth* column and *m_j1_*, *m_j2_*, and *m_j3_* are the three levels of the EC number represented by *m_j_*:

(2) $$H\left(m_{j}\right)=0.6E\left(m_{j}\right)+0.4\mathrm{Gaps}\left(m_{j}\right)$$

(3) $$E\left(m_{j}\right)=\frac{\omega_{1}E\left(m_{j1}\right)+\omega_{2}E\left(m_{j2}\right)+\omega_{3}E\left(m_{j3}\right)}{\omega_{1}+\omega_{2}+\omega_{3}}$$

(4) $$\mathrm{Gaps}\left(m_{j}\right)=\frac{\mathrm{Number}\;\mathrm{of}\;\mathrm{Gaps}}{\mathrm{Number}\;\mathrm{of}\;\mathrm{Sequences}-1}$$

where *E*(*m*_*jk*_) is the normalized entropy for the *kth* level of the *jth* column and *ω_1_*, *ω_2_*, and *ω_3_* have values of 15, 10 and 5, respectively. Then, the entropy for each column is estimated as follows,

(5) $$E\left(m_{jk}\right)=-\sum_{a}c_{jk}\left(a\right)\log_{2}p_{jk}\left(a\right)$$

where a Î {all different symbols in column j} and,

(6) $$p_{jk}\left(a\right)=\frac{c_{jk}\left(a\right)}{\sum c_{jk}\left(a\prime \right)}\text{.}$$

The probability of symbol *a* in column *jk* is the ratio of the symbol *a* counter in column *jk* and the sum of all symbol counters *a*′ (number of sequences). Gaps are also considered as a symbol [9]. It is important to mention that a column of only gaps is removed previously to objective function evaluation. Thus, the homogeneity for the whole alignment is given as,

(7) $$\mathrm{Homogeneity}=\frac{\sum_{j=1}H\left(m_{j}\right)}{\mathrm{Number}\;\mathrm{of}\;\mathrm{Columns}}\text{.}$$

### 2.7 Gap Penalty

The gaps concentration criterion is used to penalize the number of gap blocks in the whole alignment. This criterion is defined as:

(8) $$GC=\frac{{\bar{S}}_{\mathrm{GB}}}{GP}$$

where ${\bar{S}}_{\mathrm{GB}}$ is the average length of gap blocks and *GP* corresponds to the total number of individual gaps in the alignment. It is important to mention that initial and final gaps are not taken into account for the gaps penalization. If there are no gaps in the alignment, the *GC* value is zero.

This criterion serves to reward alignments where gap codifications are more concentrated, that is, where there are few larger blocks of gaps rather than blocks of smaller lengths, in contrast to Equation 4, which penalizes the number of gaps in a column. The column increment term penalizes the addition of columns into the aligned matrix and is defined as follows:

(9) $$CI=\frac{C_{0}}{C_{1}}$$

where *C*_0_ is the length of the longest sequence in the unaligned matrix and *C*_1_ corresponds to the number of columns of the aligned matrix. Thus, the defined objective function (O.F.) has to be minimized to attain better alignments.

### 2.8 Clustering

The *k*-medoids algorithm is used to cluster the sequences previously compared and included in the similarity matrix. The number of clusters is settled by using the elbow criterion and plotting the cluster quality from *k* = 2 to *k* = 100. A total of 20 replicates for each *k* value are evaluated, and the best result is selected. To define the cutoff for the clustering, the second derivative associated with this function is used, and when *k* is maximized, this value represents the greater slope, i.e., the greater difference between two *k*′ values.

## 3 Results

### 3.1 Construction of Enzymatic Step Sequences (ESS)

In order to evaluate the conserved and variable catalytic steps in metabolic pathways of the bacterium *E. coli* K-12, a collection of ESS was generated and compared using a GA. In this work, an ESS was defined as a linear collection of consecutive enzymatic reactions from a given substrate to a given product, similar to the method used for a previously proposed definition of metabolic pathways [7]. Therefore, each ESS was reconstructed by following subsequent reactions in each metabolic map. The enzymes related to each reaction were represented by using the first three levels of the EC classification to describe their general type of chemical reaction, as previously suggested [17]. In total, 452 ESS associated with 47 metabolic maps of *E. coli* K-12 were obtained [21] (see Fig. 2 and also Table SI in the supplementary material). From these, the average of sequences generated per metabolic map was 9.6 with an average length of 3.91 ± 1.1 ESS, where the map associated with pyruvate metabolism showed the highest number of ESS (145, with an average length of 9 ± 2.27 steps), probably because it represents an intersection of key pathways of energy metabolism. The second most frequent pathway corresponds to the glutamate map (39 ESS, with an average length of 5 ± 1.53 steps). Indeed, almost all maps considered in this analysis generated ESS, and the pyruvate metabolism map was also the map with the highest diversity of ESS. Finally, we found 5 pathways with only two ESS and 11 pathways with only one ESS.

Therefore, it seems that the numbers and lengths of sequences of enzymatic steps depend on the size of the metabolic map and reflect the number of alternative pathways that can be traced, beginning from the start nodes, i.e., large metabolic maps generate more ESS than small metabolic maps as a consequence of the complexity of the map. In this regard, the pyruvate metabolism is represented by a complex map, as it is the endproduct of glycolysis and the starting point for gluconeogenesis, and it can be generated by transamination of alanine. It can be converted by the pyruvate dehydrogenase complex to acetyl-CoA [6,23,25,28], which can enter the TCA cycle or serve as the starting point for the synthesis of long-chain fatty acids, steroids, and ketone bodies.

### 3.2 Significance of ESS Alignments

To evaluate the significance of the alignments, 10 random sets of ESS were constructed by shuffling the EC numbers from the real sequences, where the length and the EC composition were conserved (Figure S1). Because we were interested in evaluating the most significant scores, a threshold of fitness of 0.4 that considers “$\bar{x}-3\sigma$” was chosen, including 7.16% of the real ESS alignments. In contrast, an average of 0.34% ± 0.029 of the total random sequence alignments were below the fitness threshold of 0.4. According to these data, we expect an average of 30 ESS alignments included in our dataset to be possible false positives, suggesting that GAs infer efficiently the significant and similar sequences.

Alternatively, we assessed the consistency of the GA approach in a different scenario, one which considered a Dynamic Programing (DP) Needleman–Wunsch algorithm for the pairwise alignment of ESS. This algorithm uses the same objective function as the GA, minimizing the score of the alignment. The DP algorithm exhibits a similar (Gumbel-like) distribution as the GA fitness values, suggesting that our approach is consistent with the previous results described (Figure S2).

### 3.3 ESS Comparisons Identify Groups of Similar Reactions in Different Pathways

In order to evaluate the similarity of the metabolic maps for the bacterium *E. coli* K-12, 452 ESS were used to carry out all-against-all pairwise alignments. Therefore, the GA previously described was applied based on the objective function (O.F.), with which we evaluated the entropy per column using a population of 100 chromosomes, a 1% mutation rate and 90% crossover rate. Mutation is applied per individual and it means that an individual will be mutated each generation. Therefore, an individual in the population is a particular possible solution to the ESS alignment. The algorithm was concluded when 20 generations were reached with no changes in the objective function. Ten replicates of the GA were performed, and the best result was chosen. The GA evaluates the alignment by using a normalized entropy-based function defined above in Eq. (1) (see the Methods section). Since the fitness value is a measure of the entropy for each column in the alignment, a value near 0 corresponds to an alignment with homogeneous columns, i.e., columns containing similar EC numbers. Conversely, values near 1 correspond to alignments with columns that are less homogeneous, i.e., columns containing dissimilar EC numbers. Therefore, the fitness value is a measure of how similar two sequences are. Based on similarity values from the all-against-all comparisons, a matrix that considered the similarity (fitness) values of 452 vs 452 sequences was constructed. This matrix was posteriorly analyzed by using the *k*-medoids algorithm and identified groups with similar ESS that may share similar catalytic properties. To determine the number of clusters (*k*) that could be generated, the elbow criterion was used. In addition, diverse *k* values were used to construct different clusters: *k* = 21, 27, and 34, as these exhibited the higher peaks in the analysis plot. As a criterion to discriminate between the clustering at *k* = 21, 27, and 34, the clusters were depurated, eliminating any sequence whose mean fitness with the rest of the sequences of its cluster was above 0.4. After depuration, 93, 71, and 37 sequences were eliminated for *k* = 21, 27, and 34, respectively. In this way, we selected a *k* value of 34 to minimize the number of sequences eliminated during depuration.

**Fig. 4:**
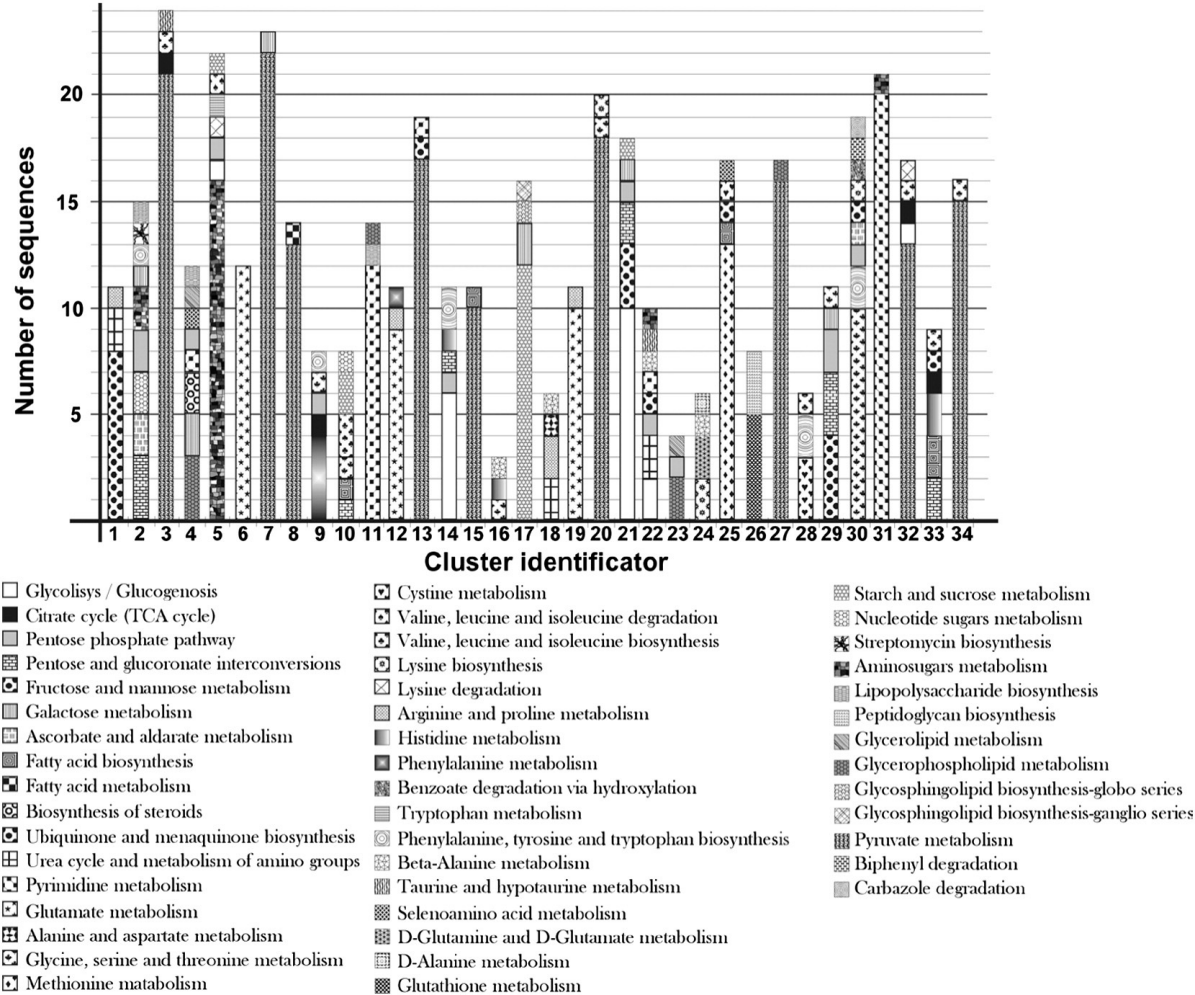
Distribution of ESS among the 34 identified clusters. Bars represent the number of sequences per cluster.

From this analysis, similar ESS associated with diverse pathways were clustered together. For instance, the ESS constructed from the pyruvate map were differentially clustered in nine groups with sequences belonging to different pathways, including those for the citrate cycle (TCA cycle), glycolysis/gluconeogenesis, and lysine, glycine, serine, and threonine metabolism (Fig. 4). This finding correlates with the fact that pyruvate is the endproduct at which diverse metabolic pathways converge, including glycolysis, gluconeogenesis, and alanine metabolism, among others. Based on these results, we suggest that similar catalytic processes are required to metabolize different compounds, to converge at a compound, probably by diverse recruitment events, where the catalytic activity is preferentially coupled, as previously noticed using as the comparative the first two digits associated with EC numbers [2,8,19]. In addition, some clusters are comprised almost exclusively of sequences belonging to pyruvate metabolism, such as clusters 7, 8, 13, and 15, which implies that some ESS have probably been duplicated to increase this metabolic pathway and its product.

Alternatively, cluster 4 contains sequences from different maps related to lipid metabolism, including lipopolysaccharide, glycerolipid, glycerophospholipid, and steroid metabolism, pyrimidine metabolism, selenium amino acids, the pentose phosphate pathway, and galactose metabolism. The similar catalytic activities in all these pathways suggest the phenomenon of recruitment events. Finally, cluster 23 contains exclusively ESS from the glycerolipid and glycerophospholipid metabolic pathways, suggesting a common origin shared by these two metabolic pathways. These results correlate with the origin of fatty acid metabolism by gene duplication [8]. Thus, an ancestral pathway catalyzed by both fatty acid degradation and biosynthesis could have originated in a first step. The direction of this ancestral pathway would be dependent on the acyl carriers and fatty acids available, providing evidence of the existence of two different pathways [8,15,19].

Similar results were observed in the ESS included in clusters 18, 24, 22, 25, and 30, which included reactions from diverse amino acid metabolism pathways, whereas clusters 2, 11, 17, 21, 23, and 29 included ESS from carbohydrate metabolism. In particular, clusters 17 and 21 included ESS belonging to galactose, starch, and sucrose metabolic pathways; cluster 12 included ESS from glycosphingolipid biosynthesis; and cluster 21 included ESS from the galactose, pentose, sucrose, mannose, and glycolysis/gluconeogenesis pathways. All these findings suggest that the assimilation of these carbon sources involved identical enzymatic processes that may have arisen from recruitment events, according to the *Patchwork Model* of evolution.

### 3.4 Multiple Sequence Alignments (MSA) of Similar ESS

**Fig. 5:**
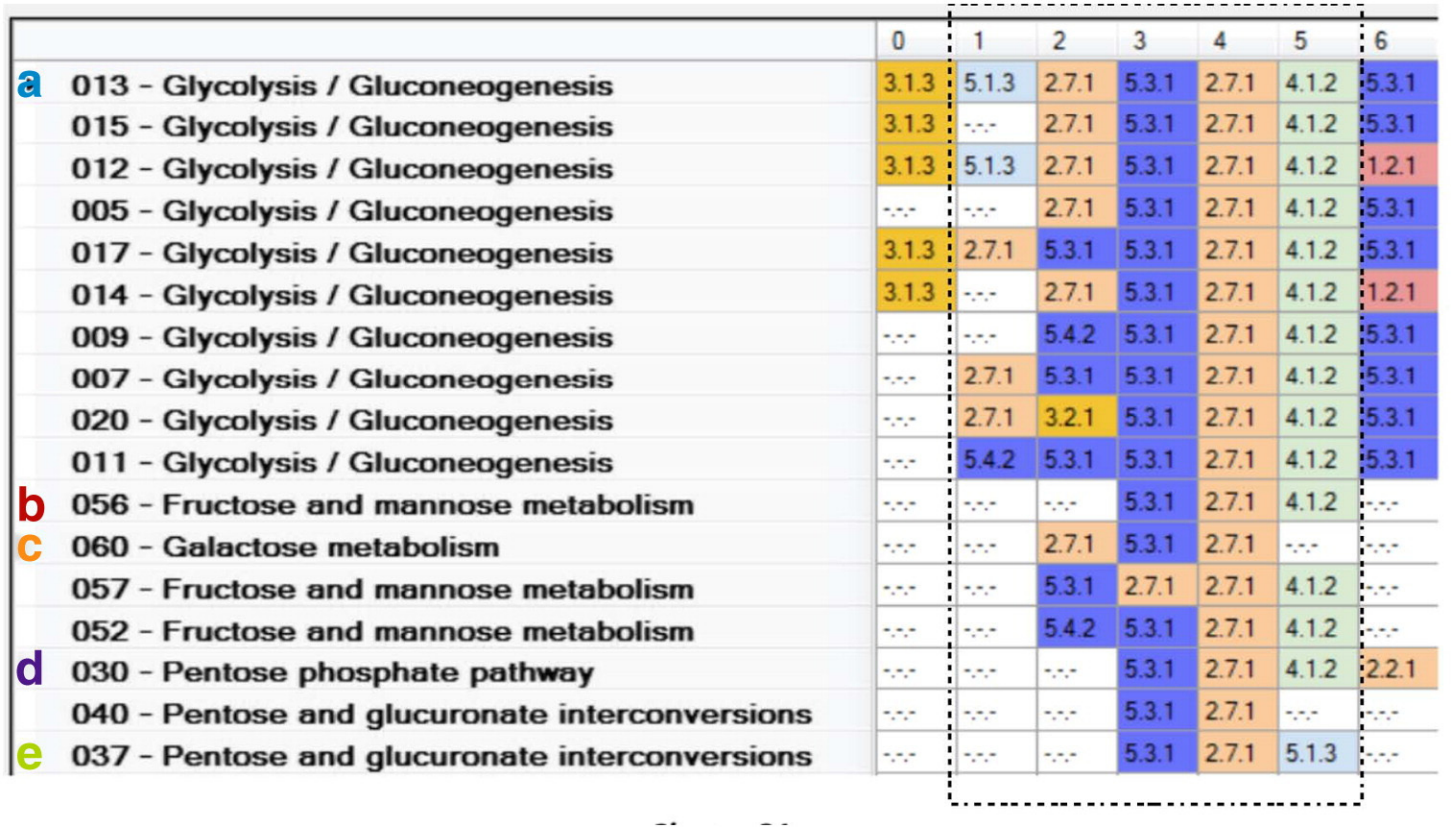
MSA of the ESS included in cluster 21.

In order to maximize the identification of functional similarities among sequences included in a common cluster, MSAs were performed using a progressive GA alignment and an objective function based on entropy. This function considered three main factors, determined empirically, to evaluate the enzymatic number level (15, 10, or 5). The progressive GA works as follows: the ESS are sorted according to their similarity values, and this order is used to guide the sequence alignment, considering the more similar pair and adding a third one, and so on. This algorithm increases column homogeneity among the sequences aligned and penalizes the insertion of gaps; hence, the fitness function has to be minimized to obtain better alignments. From this analysis, in Fig. 5 we show the MSA of cluster 21. Cluster 21 contains 17 ESS from five different maps related to carbohydrate metabolism: glycolysis/gluconeogenesis, fructose and mannose metabolism, galactose metabolism, the pentose phosphate pathway, and pentose and glucoronate interconversions. In this cluster, columns 3 and 4 are identical in almost all the sequences, suggesting a core of enzymatic reactions conserved among all these pathways and a common metabolic mechanism used to metabolize these carbohydrates. This *core* of ESS includes an isomerization (EC 5.3.1) reaction followed by a phosphorylation (EC 2.7.1) reaction, suggesting that these reactions are ancestral to all these pathways or that they were recruited in small catalytic modules [8], whereas surrounding reactions could be added to increase the other metabolic pathways.

**Fig. 6:**
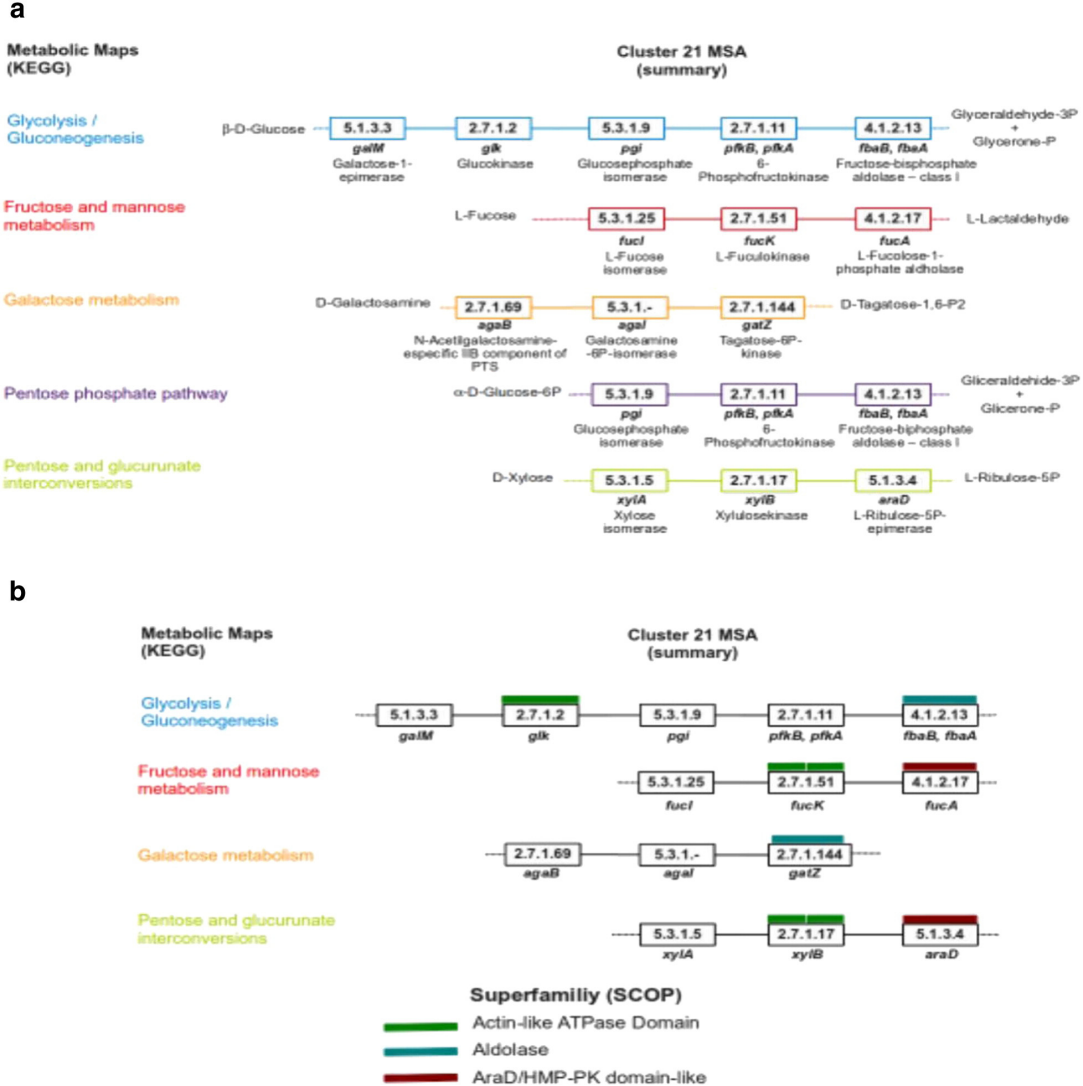
Structural domain assignment according to the Superfamily database for proteins aligned in cluster 21. In panel A, all of the enzymes in the aignment were mapped in the corresponding metabolic map. Panel B are the results of the Superfamily domain assignations. Only the similar domains are indicated.

Nevertheless, it is expected that similar enzymatic steps found in different metabolic maps actually represent different proteins. Therefore, to examine this phenomenon, five ESS were selected from cluster 21 that represented each of the five metabolic maps, and the information for protein sequences associated with each EC number was obtained. Based on this approach, the ESS from the glycolysis/gluconeogenesis, fructose and mannose metabolism, galactose metabolism, and pentose and glucoronate metabolism pathways, representing different enzymes, as shown in Fig. 6, were compared. Sequences representing the pentose phosphate pathway are a subset of the glycolysis/gluconeogenesis pathway; this is a consequence of how KEGG organizes and represents graphically the metabolic maps. Therefore, the proposed method is able to identify similar ESS assigned to different metabolic contexts.

Based on these data, it was suggested that the similar enzymatic steps identified by this strategy may share a common origin, i.e., they have been recruited from different pathways. To evaluate the probable common origin of these enzymes, their amino acid sequences were analyzed using the domain identification system based on Superfamily database assignations [20]. In brief, Superfamily is a database devoted to identification of structural domains at the level of the Superfamily. This definition is based on the SCOP database classification [12]. Therefore, two proteins share a common ancestor if they contain domains belonging to the same Superfamily. In Fig. 6 an illustrative example is shown, with only the structural domains identified for at least two enzymes in the alignment of enzymatic steps previously described. From this analysis, three different structural domains were identified as repeated in at least two enzymes in the alignment of cluster 21: the actin-like ATPase domain, the aldolase domain, and AraD/HMP-PK domain-like. Therefore, the enzymatic steps common to the pathways included in cluster 21 may have been recruited posterior to an event of gene duplication. In addition, similar enzymatic steps identified in the glycolysis/gluconeognesis, fructose and mannose metabolism, and pentose phosphate and glucuronate interconversion metabolic maps (Glk, FucK, and XilB, respectively) and the different evolutionary origins for some of their protein sequences suggest an example of *Patchwork Evolution* [16], in which the ancestor of these enzymes might be associated with diverse duplication events with posterior recruitment to different metabolic pathways. The catalytic activity associated with EC 2.7.1 was mainly recruited to perform a link between two isomerization reactions with the EC numbers 5.x.x.

Based on this analysis we identified that three proteins (FbaB, FbaA, and GatZ) share a common structural domain, identified by superfamily, the aldolase domain, and two else (FucA and AraD) the AraD/HMO-PK domain-like, which catalyze dissimilar reactions. These proteins might represent two cases of divergent evolution, in which two enzymes from a common ancestor diverged at different functions. Therefore, according to our data, it is clear that convergent evolution events are also frequently associated with enzymes devoted to metabolism; for instance, four nonhomologous enzymes (Pgi, FucI, AgaI, and XylA) catalyze the isomerization reaction 5.3.1.x, conserved in all the sequences of the cluster. This finding correlates with previous reports that showed that catalytic convergent evolution is a common phenomenon [13,24,25].

Finally, it is interesting that in the case of the sequences representing the fructose and mannose metabolism pathways and the pentose and glucoronate metabolism pathways, the FucK–FucA and XylB–AraD enzymatic steps are catalyzed by enzymes sharing a common structural domain, suggesting that the approach described here is able to identify probable patchwork events as well as duplication and convergent enzymes associated with the evolution of a microorganisms's metabolism.

## 4 Discussion and Conclusions

In general, two main approaches to compare metabolic pathways have been proposed. In one approach entails methods based on the alignment of complete or fragmented networks [3,10,18,22,29], that take into account the structure of the metabolic network and integrate more information about the system. However, an increase in complexity also increases the computational cost, making it more difficult the possibility to create multiple alignments. Via the other approach, entailing methods based on the alignment of linear pathways [7,27]; and that was utilized in this work, the complexity of the comparisons is reduced, and they can be executed and analyzed relatively easily.

In this regard, sequence alignment is a problem in bioinformatics where it is important to identify conserved regions in a set of sequences, and as a consequence, to find the differences between them based on a criterion to assess the quality of an alignment. Thus, a GA was proposed in order to optimize the quality criterion of potential alignments considering maximization of homogeneity and, at the same time, minimization of gaps. The proposed GA used a variable binary representation of individual enzymes (potential alignments) where 1 represents EC numbers and 0 indicates the gaps.

In the present work, the strategy to transform enzymatic reaction sequences from a metabolic pathway allowed us to implement a sequence alignment method in an efficient and easy fashion, where regions sharing a similar succession of EC steps were identified, suggesting common catalysis that is not easy to identify when using traditional computational tools. The progressive GA proposed method has shown efficient results, providing good alignments after less than 100 iterations. These results allow us to make comparative studies of metabolic pathways to elucidate the functions of newly discovered pathways, increase our understanding of evolutionary traits, and identify potential missing pathway elements. It is interesting that some of the enzymatic steps common to various sequences may, indeed, represent the same reactions, such as the first 10 sequences in Fig. 6, which have a different representation of the same region of the map of the glycolysis/gluconeogenesis pathway, although they represent the same reaction sequence steps. Moreover, these sequences are not identical, and they differ mainly in the beginnings or in the ends of the sequences. This implies that the proposed method introduced in this paper may have the capability to show alternative metabolic pathways not previously described.

In summary, we consider that the strategy described in this work allowed us to identify similar ESS from different metabolic maps, as shown in Figs. 5 and 6. Thus, maps associated with the metabolism of similar compounds also contain similar ESS, reinforcing the *Patchwork Model* for studying the evolution of metabolism in *E. coli* K-12, where similar consecutive enzymatic steps may have different origins, in agreement with biochemical restrictions in enzymatic recruitment [2,8], an observation that can be expanded to other organisms for which metabolism information is available.

## Appendix A Supplementary Data

Supplementary material **Figure S1**. **Cumulative histogram of the fitness of the pairwise alignments of random generated ESSs**. In order to assess the statistical significance of the ESS pairwise alignments, 10 random sets were generated containing the same composition and length size than the original ESSs. The figure shows the mean ± standard deviation of the random alignments. The real data has an overrepresentation of scores below 0.4 that corresponds to the fitness values of the alignments of similar ESSs. **Figure S2**. **Histogram of the fitness (scores) of the pairwise alignments of ESSs**. The comparison between the scores obtained by GA and Dynamic Programing (DP) algorithms are shown. In order to asses the consistence of the GA we implement a DP algorithm that creates and evaluates the ESS alignments using the same Objective Function used by the GA. This algorithm were used to repeat the all against all pairwise alignments previously generated with the GA. The results show that both algorithms generate similar distributions.

## References

[1] Armenta-Medina D., Perez-Rueda E., Segovia L. (2011). Identification of functional motions in the adenylate kinase (ADK) protein family by computational hybrid approaches. Proteins.

[2] Armenta-Medina D., Segovia L., Perez-Rueda E. (2014). Comparative genomics of nucleotide metabolism: a tour to the past of the three cellular domains of life. BMC Genomics.

[3] Ay F., Kellis M., Kahveci T. (2011). SubMAP: aligning metabolic pathways with subnetwork mappings. J Comput Biol.

[4] Caetano-Anolles G., Kim H.S., Mittenthal J.E. (2007). The origin of modern metabolic networks inferred from phylogenomic analysis of protein architecture. Proc Natl Acad Sci U S A.

[5] Caspi R., Altman T., Billington R. (2014). The MetaCyc database of metabolic pathways and enzymes and the BioCyc collection of Pathway/Genome Databases. Nucleic Acids Res.

[6] Chandrasekhar K., Wang J., Arjunan P. (2013). Insight to the interaction of the dihydrolipoamide acetyltransferase (E2) core with the peripheral components in the *Escherichia coli* pyruvate dehydrogenase complex via multifaceted structural approaches. J Biol Chem.

[7] Chen M., Hofestadt R. (2004). PathAligner: metabolic pathway retrieval and alignment. Appl Bioinformatics.

[8] Diaz-Mejia J.J., Perez-Rueda E., Segovia L. (2007). A network perspective on the evolution of metabolism by gene duplication. Genome Biol.

[9] Durbin R., Eddy S., Krogh A., Mitchison G. (1998). Biological sequence analysis: probabilistic models of proteins and nucleic acids.

[10] Flannick J., Novak A., Srinivasan B.S., McAdams H.H., Batzoglou S. (2006). Graemlin: general and robust alignment of multiple large interaction networks. Genome Res.

[11] Forst C.V., Schulten K. (2001). Phylogenetic analysis of metabolic pathways. J Mol Evol.

[12] Fox N.K., Brenner S.E., Chandonia J.M. (2013). SCOPe: structural classification of proteins—extended, integrating SCOP and ASTRAL data and classification of new structures. Nucleic Acids Res.

[13] Gherardini P.F., Wass M.N., Helmer-Citterich M., Sternberg M.J. (2007). Convergent evolution of enzyme active sites is not a rare phenomenon. J Mol Biol.

[14] Hernandez-Montes G., Diaz-Mejia J.J., Perez-Rueda E., Segovia L. (2008). The hidden universal distribution of amino acid biosynthetic networks: a genomic perspective on their origins and evolution. Genome Biol.

[15] Hla T. (2005). Genomic insights into mediator lipidomics. Prostaglandins Other Lipid Mediat.

[16] Jensen R.A. (1976). Enzyme recruitment in evolution of new function. Annu Rev Microbiol.

[17] Klein C.C., Cottret L., Kielbassa J., Charles H., Gautier C., Ribeiro de Vasconcelos A.T. (2012). Exploration of the core metabolism of symbiotic bacteria. BMC Genomics.

[18] Kolar M., Meier J., Mustonen V., Lassig M., Berg J. (2012). GraphAlignment: Bayesian pairwise alignment of biological networks. BMC Syst Biol.

[19] Light S., Kraulis P., Elofsson A. (2005). Preferential attachment in the evolution of metabolic networks. BMC Genomics.

[20] Oates M.E., Stahlhacke J., Vavoulis D.V. (2014). The SUPERFAMILY 1.75 database in 2014: a doubling of data. Nucleic Acids Res.

[21] Okuda S., Yamada T., Hamajima M., Itoh M., Katayama T., Bork P. (2008). KEGG Atlas mapping for global analysis of metabolic pathways. Nucleic Acids Res.

[22] Pinter R.Y., Rokhlenko O., Yeger-Lotem E., Ziv-Ukelson M. (2005). Alignment of metabolic pathways. Bioinformatics.

[23] Reed L.J., Hackert M.L. (1990). Structure–function relationships in dihydrolipoamide acyltransferases. J Biol Chem.

[24] Ryan A., Wang C.J., Laurieri N., Westwood I., Sim E. (2011). Reaction mechanism of azoreductases suggests convergent evolution with quinone oxidoreductases. Protein Cell.

[25] Schilling S., Wasternack C., Demuth H.U. (2008). Glutaminyl cyclases from animals and plants: a case of functionally convergent protein evolution. Biol Chem.

[26] Silvela J., Portillo J. (2001). Breadth-first search and its application to image processing problems. IEEE Trans Image Process.

[27] Tohsato Y., Matsuda H., Hashimoto A. (2000). A multiple alignment algorithm for metabolic pathway analysis using enzyme hierarchy. Proc Int Conf Intell Syst Mol Biol.

[28] Wang J., Nemeria N.S., Chandrasekhar K. (2014). Structure and function of the catalytic domain of the dihydrolipoyl acetyltransferase component in *Escherichia coli* pyruvate dehydrogenase complex. J Biol Chem.

[29] Wernicke S., Rasche F. (2007). Simple and fast alignment of metabolic pathways by exploiting local diversity. Bioinformatics.

[30] Yang Q., Sze S.H. (2007). Path matching and graph matching in biological networks. J Comput Biol.

