# Supplementary material for: Comparison of Metabolic Pathways in Escherichia coli by Using Genetic Algorithms
Source: Comput Struct Biotechnol J. 2015 Apr 9;13:277–85. doi: 10.1016/j.csbj.2015.04.001 (PMC4423528; doi:10.1016/j.csbj.2015.04.001)
Supplement: Supplementary material — Figure S1. Cumulative histogram of the fitness of the pairwise alignments of random generated ESSs. In order to assess the statistical significance of the ESS pairwise alignments, 10 random sets were generated containing the same composition and length size than the original ESSs. The figure shows the mean ± standard deviation of the random alignments. The real data has an overrepresentation of scores below 0.4 that corresponds to the fitness values of the alignments of similar ESSs. Figure S2. Histogram of the fitness (scores) of the pairwise alignments of ESSs. The comparison between the scores obtained by GA and Dynamic Programing (DP) algorithms are shown. In order to asses the consistence of the GA we implement a DP algorithm that creates and evaluates the ESS alignments using the same Objective Function used by the GA. This algorithm were used to repeat the all against all pairwise alignments previously generated with the GA. The results show that both algorithms generate similar distributions. [file mmc1.ppt]

## Slide 1
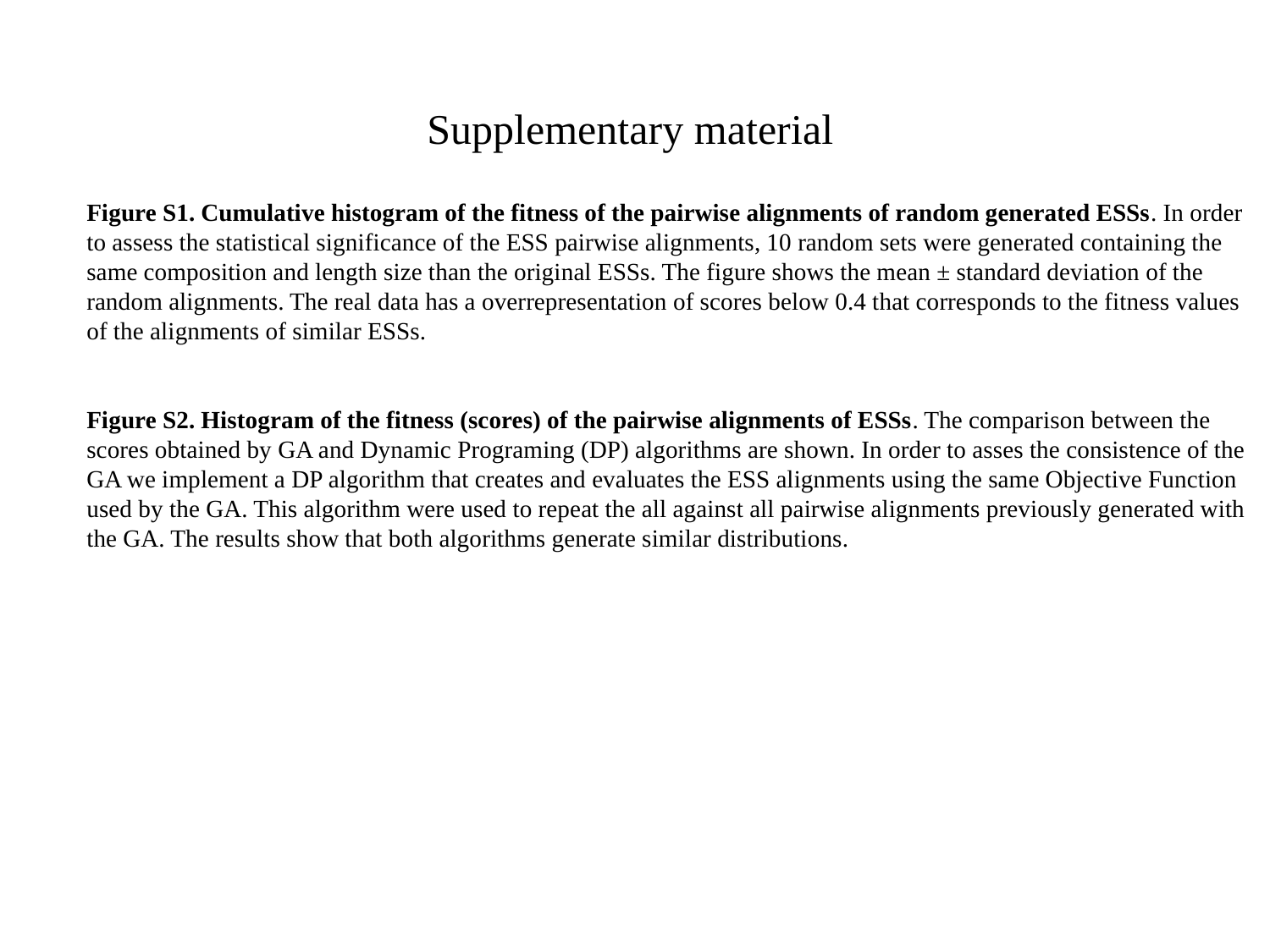

Supplementary material
Figure S1. Cumulative histogram of the fitness of the pairwise alignments of random generated ESSs. In order to assess the statistical significance of the ESS pairwise alignments, 10 random sets were generated containing the same composition and length size than the original ESSs. The figure shows the mean ± standard deviation of the random alignments. The real data has a overrepresentation of scores below 0.4 that corresponds to the fitness values of the alignments of similar ESSs.
Figure S2. Histogram of the fitness (scores) of the pairwise alignments of ESSs. The comparison between the scores obtained by GA and Dynamic Programing (DP) algorithms are shown. In order to asses the consistence of the GA we implement a DP algorithm that creates and evaluates the ESS alignments using the same Objective Function used by the GA. This algorithm were used to repeat the all against all pairwise alignments previously generated with the GA. The results show that both algorithms generate similar distributions.

## Slide 2
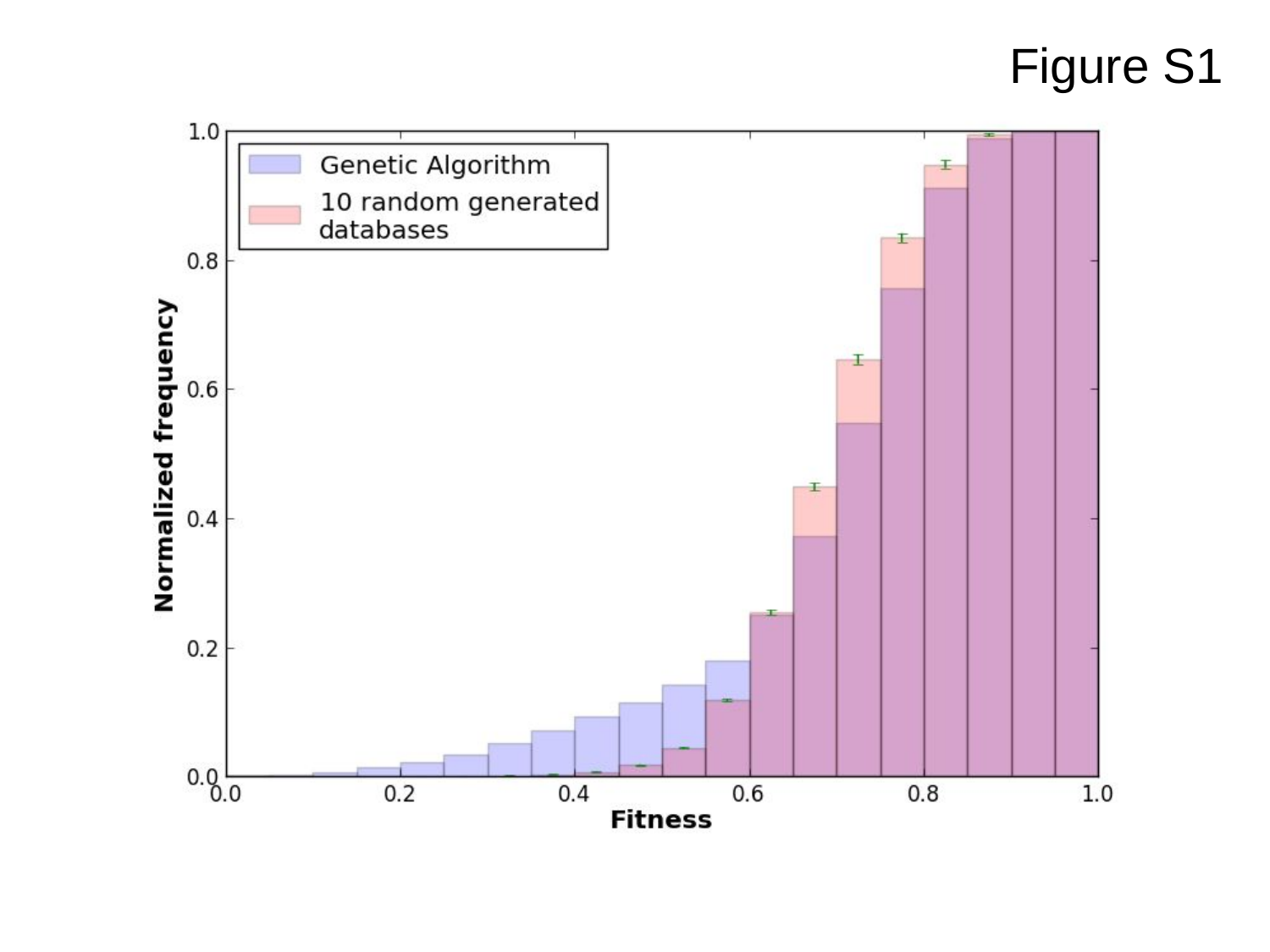

Figure S1

## Slide 3
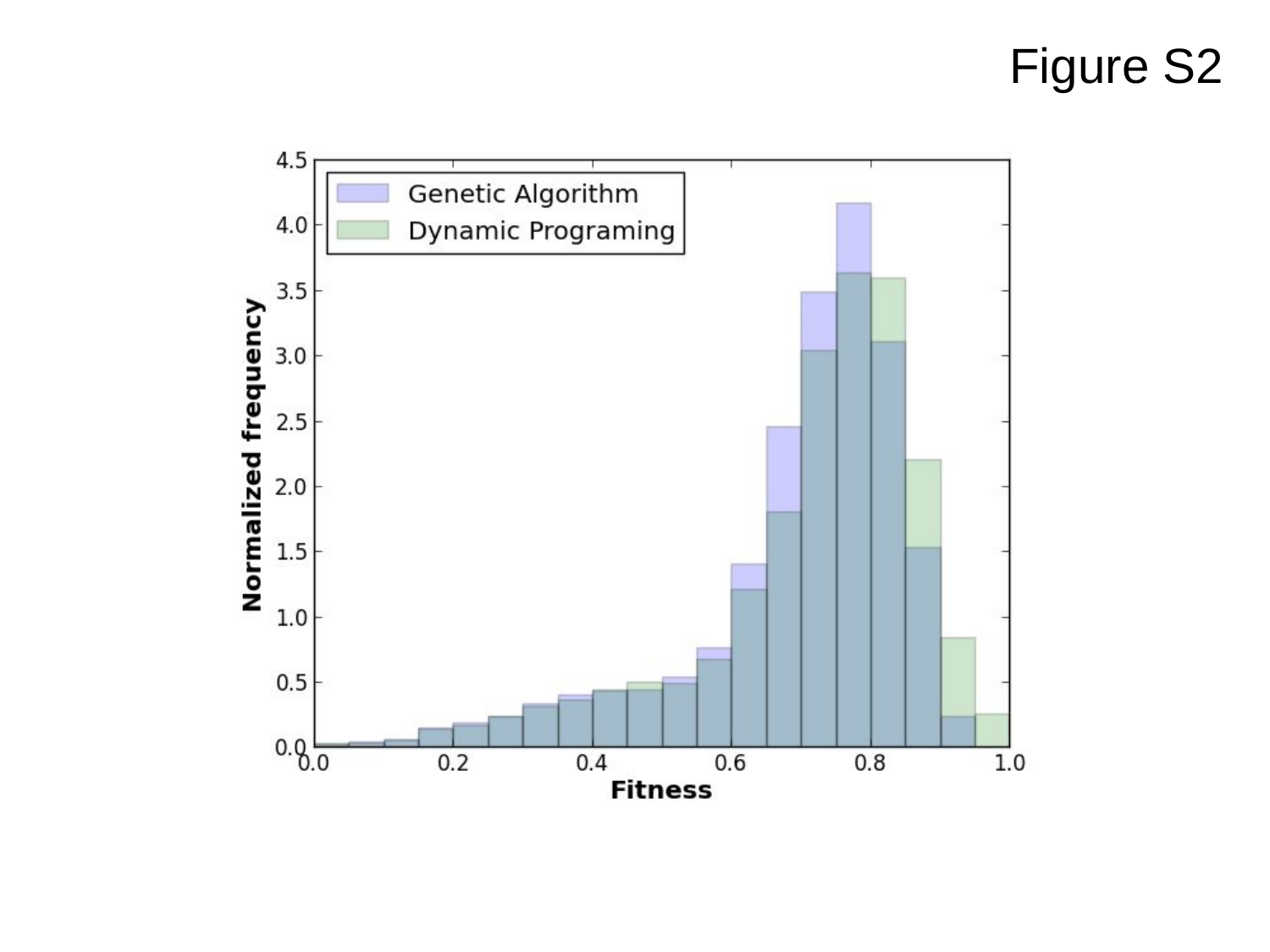

Figure S2

## Slide 4
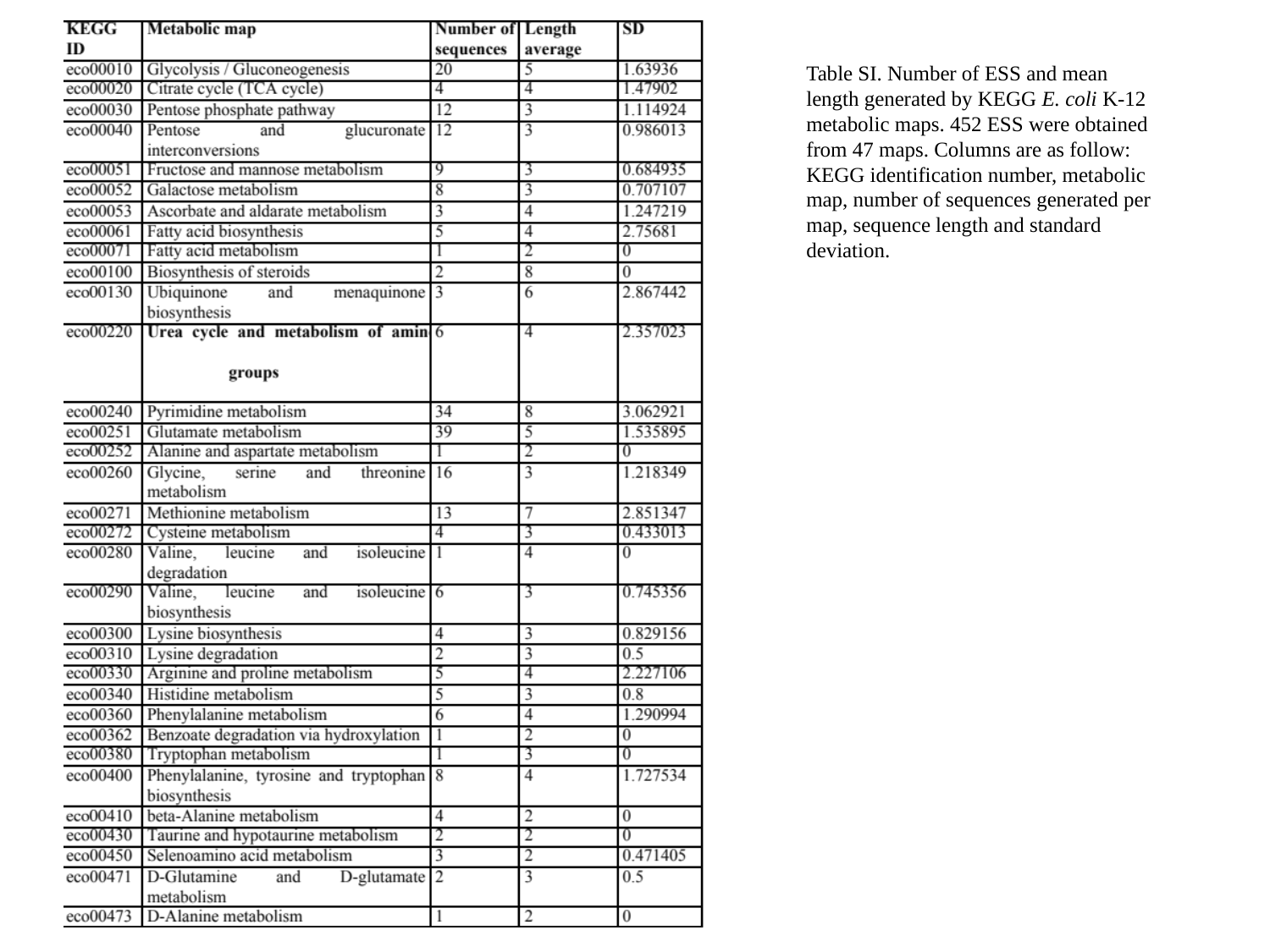

Table SI. Number of ESS and mean length generated by KEGG E. coli K-12 metabolic maps. 452 ESS were obtained from 47 maps. Columns are as follow: KEGG identification number, metabolic map, number of sequences generated per map, sequence length and standard deviation.

## Slide 5
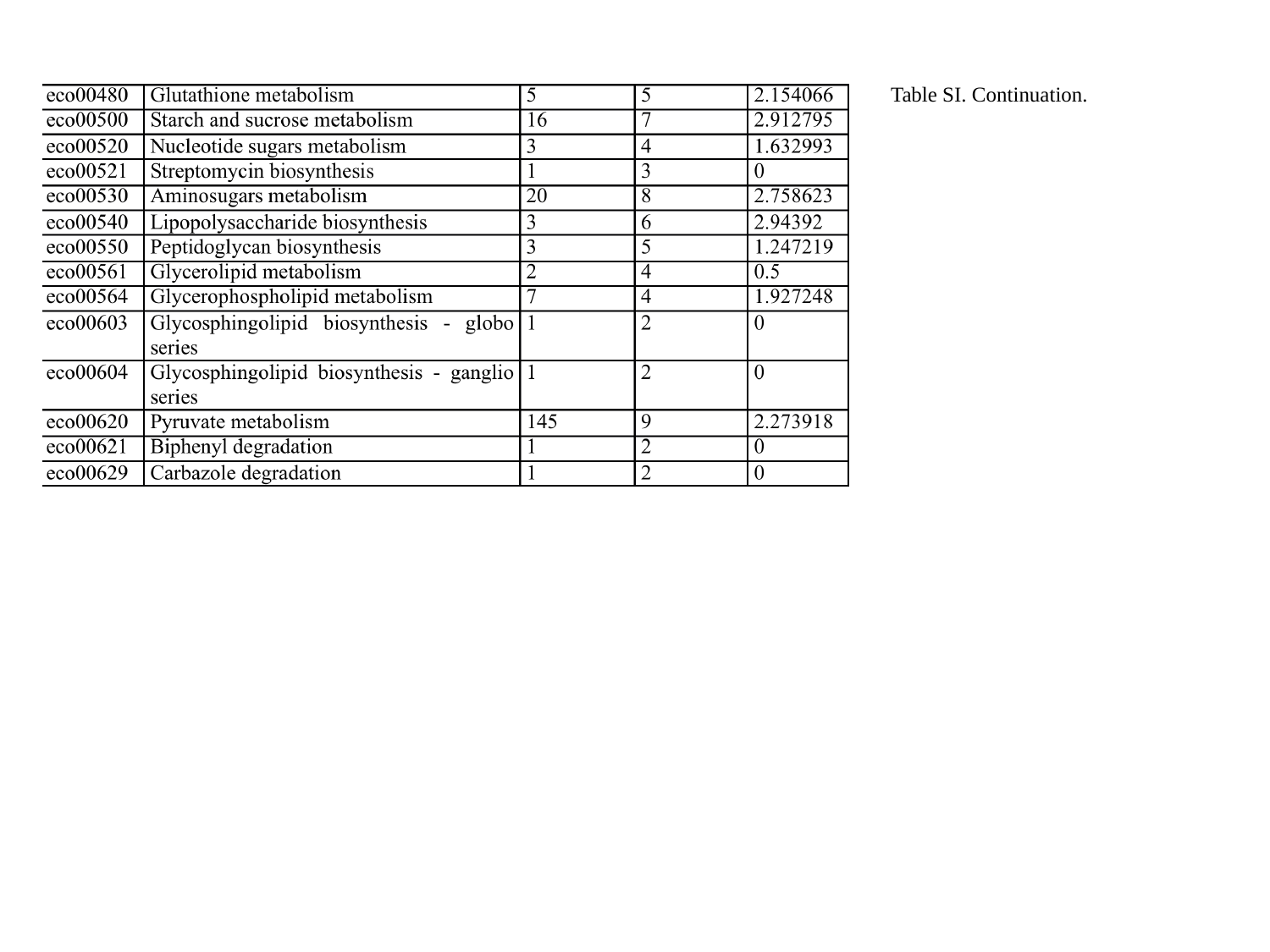

Table SI. Continuation.
